# Multivariate analysis between environmental factors and fruit quality of citrus at the core navel orange-producing area in China

**DOI:** 10.3389/fpls.2024.1510827

**Published:** 2024-12-02

**Authors:** Xiaoxuan Yu, Chao Du, Xiaojun Wang, Fengying Gao, Jing Lu, Xinyue Di, Xia Zhuang, Chen Cheng, Fengxian Yao

**Affiliations:** ^1^ School of Geography and Environmental Engineering, Gannan Normal University, Ganzhou, China; ^2^ National Navel Orange Engineering Research Center, School of Life Sciences, Gannan Normal University, Ganzhou, China

**Keywords:** fruit quality, soil factor, meteorological factor, topographic factor, model effect

## Abstract

Gannan is the largest navel orange production area in China. Most studies have primarily focused on the effects of either soil or topographic factors on the quality of navel oranges. However, there has been a lack of research exploring the relationship between navel orange quality and multiple environmental factors (meteorological, topographic, and soil). This study focused on Gannan navel oranges, selecting standard orchards in the core navel orange-producing area as the research region. It employed the Partial Least Squares Regression (PLSR) method to investigate the extent of the impact of environmental factors on fruit quality. The results indicated that the effect of soil factors on fruit shape and fruit flavor was greater than that of meteorological and topographic factors in the Gannan area. And the fruit peel is more uniformly influenced by environmental factors. Based on the degree of impact of various environmental factors, multiple regression equations for fruit quality have been established to identify the optimal conditions conducive to the comprehensive development of Gannan navel oranges. These findings help determine the optimal planting areas for Gannan navel oranges, providing practical evidence for the future development of navel oranges.

## 1 Introduction

Fruit qualities are mainly determined by environmental nutrition and cultivation techniques (Morales Alfaro et al., 2023), which strongly affect the quantity of Gannan navel orange (García-Muñoz et al., 2021). Most of the related works focused on the effect of soil factors on fruit qualities (Silva-De Paula et al., 2022; Shirgure and Srivastava, 2022). Some researchers reported the influence on fruit qualities by meteorological and topographic factors (Panigrahi et al., 2021; Orhan, 2021). However, no comprehensive research has systematically studied the relationships between navel orange qualities and environmental factors (soil, meteorological, and topographic factors). The Gannan navel orange is a cultivar of sweet orange within the genus Citrus, known for its rich nutrient content (Gao et al., 2019). The quality of navel oranges can be evaluated by shape (horizontal diameter, vertical diameter, shape index), peel (luminance, green-red difference, yellow-blue difference, hue angle), and flavor (soluble solid, titratable acid, solid-acid ratio, Vitamin C), meanwhile, the weight of individual navel oranges can indicate overall yield.

According to previous studies, among the factors affecting fruit qualities, meteorological, topographic, and soil factors are crucial environmental determinants of navel orange qualities. Topographic factors are associated with the growth rate of navel orange fruit trees. The large slope is unsuitable for cultivating citrus crops. Meanwhile, climatic changes can impact the taste and yield of citrus fruits (Dong et al., 2024). The variations in average temperature and precipitation can alter the water table, potentially damaging fruit trees and fruits (Karamidehkordi et al., 2023). Importantly, plants cannot thrive without soil. Soil serves as the essential medium for water and nutrient uptake, as well as material and energy exchange in plants (Ma et al., 2024). The physical and chemical properties of soil simultaneously affect fruit qualities (Dong et al., 2021; Zhou et al., 2021; Jin et al., 2022; Xia et al., 2022; Santiago et al., 2023).

Ganzhou City in Jiangxi Province boasts the world’s largest annual output of navel oranges and is renowned as the “Orange Hometown of the World”. Xinfeng and Anyuan counties serve as the core production areas for Gannan navel oranges. In 2023, their output reached 260,000 tons and 210,000 tons, respectively, ranking them among the top three navel orange producers in Ganzhou City (Liu et al., 2024). This study aimed to identify the primary environmental factors (meteorological, topographic, and soil factors) influencing the quality characteristics of Gannan navel oranges, and to propose an optimization strategy for these factors to enhance the quality of Gannan navel oranges. The findings from this study will inform strategic regional planning and cultivation practices for Gannan navel oranges.

## 2 Materials and methods

### 2.1 Study area

Xinfeng County and Anyuan County, situated in the southern part of Jiangxi Province, are renowned as the core navel orange-producing regions in Gannan, covering a total area of 2,878 square kilometers. The core production area of the Gannan navel orange (24° 52 ′ N ~ 25° 36 ′ N, 114° 34 ′ E ~ 115° 37 ′ E) is situated within the subtropical monsoon climate zone, characterized by a warm and humid climate, with an average annual temperature stabilized at about 19.8 °C, and abundant annual precipitation reaching 1580.2 mm. These conditions provide a favorable climate for the growth of navel oranges. In addition, the core production areas of the Gannan navel orange are primarily hilly, featuring moderate elevations and efficient drainage, which collectively enhance the growth and development of the navel oranges. Most of its soil is red and acidic, characteristic of the typical red soil distribution found in southern China. These superior climate, topography, and soil conditions establish it as a prime area for navel orange cultivation in China. As of 2023, the annual production of Gannan navel orange in the core production area has exceeded 400,000 tons (Liu et al., 2024).

### 2.2 Fruit sampling and quality analysis

**Figure 1:**
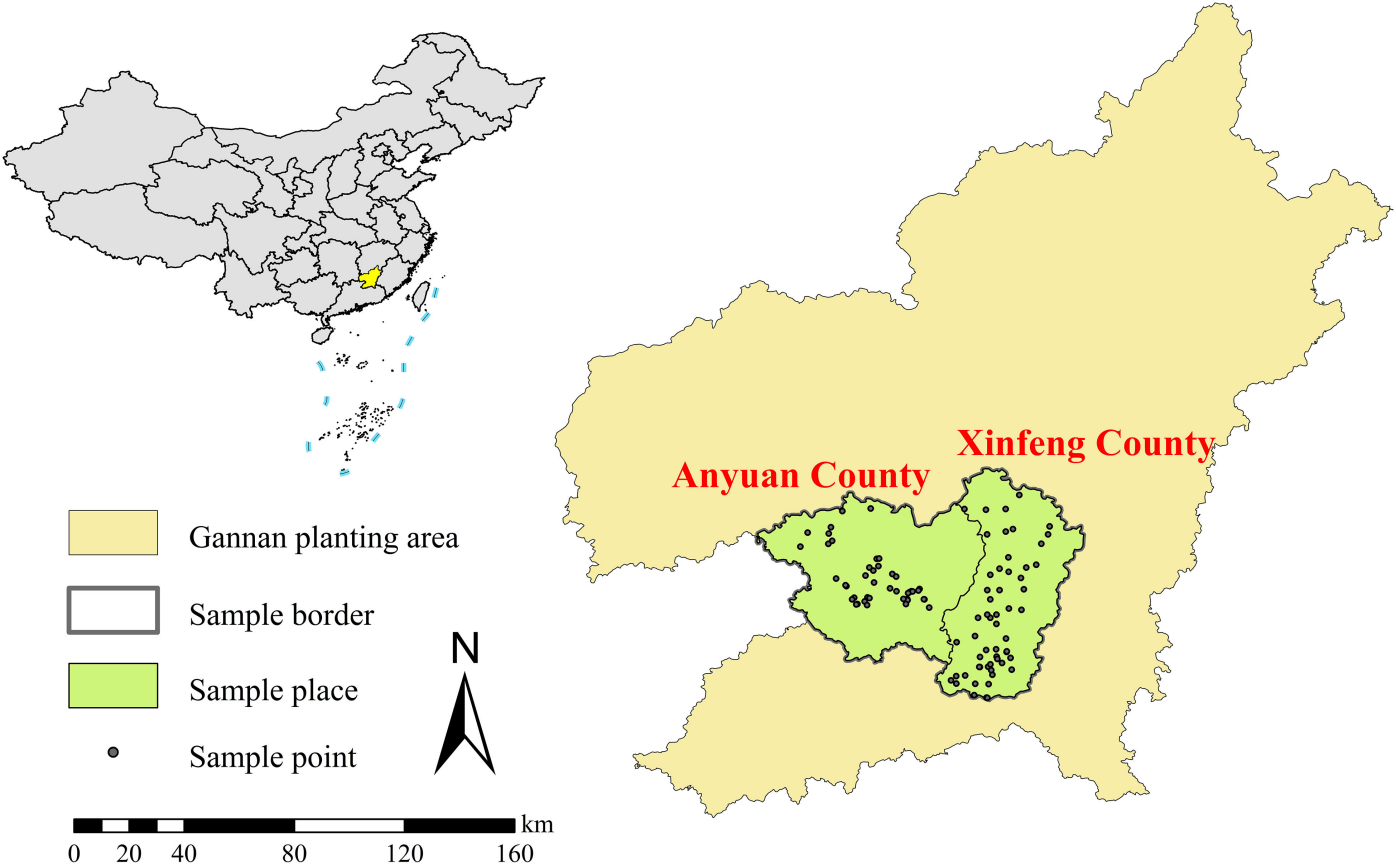
Geographical locations of sampling orchards.

In this study, 99 standard navel orange orchards of varying ages in the fruiting stage were selected in the core production area of the Gannan navel orange. Data collection occurred in November 2023, during the ripening phase of the navel oranges. The locations of the collection points are illustrated in **Figure 1**. Three navel orange trees with uniform shape and growth characteristics were selected from each planting area. Five fruits were randomly sampled from around the tree crown. Soil samples were collected from a depth of 0 to 40 cm, 10 cm inside the drip line of the tree crown while avoiding fertilizer holes. Soil and fruit samples from the same sampling point were individually mixed and then combined into a composite sample using the 4-part method.

The mass of each fruit (in grams) was measured using an electronic balance, and the fruit size and vertical and horizontal measurements were determined using a digital Vernier caliper. The fruit shape index was calculated by dividing the horizontal diameter by the vertical diameter. Peel luminance (L*), green-red difference (a*), yellow-blue difference (b*), and hue angle (H) were assessed with a CR-400 handheld colorimeter. Luminance (L*) represents the brightness of the pericarp; the higher the L* value, the brighter the surface. The a* value indicates the red-green color difference of the pericarp, with a change from -a* to +a* signifying a decrease in green and an increase in red. The b* value denotes the yellow-blue color difference of the pericarp, where a shift from -b* to +b* indicates a decrease in blue and an increase in yellow.

The total soluble solid (TSS) content was measured by Handheld Refractometer (ATAGO Classic HHR-2N). The titratable acid (TA) content was determined by titration with a 0.1M standard solution of sodium hydroxide (Treeby et al., 2007). The solid-acid ratio (TSS/TA) was calculated based on the values of TSS and TA. Vitamin C content was determined by the 2,6-dichloroindophenol titration method (Wu et al., 2024).

### 2.3 Soil factors measurement

According to standard agricultural chemistry procedures for soil analysis (Lu, 1999), the measurement of soil pH was conducted using a soil-to-water ratio of 1:2.5. To determine the soil organic matter (SOM), this study used the K_2_CrO_4_-H_2_SO_4_ oil-bath heating method. The cation exchange capacity (CEC) was evaluated via the 1 M CH_3_COONH_4_ leaching method at pH 7.0. The soil clay content was determined by using the hydrometer method. Following standard soil agrochemical analysis procedures (Bao, 2000), the quantification of hydrolyzed N was conducted utilizing the alkaline hydrolysis-diffusion method. Total P was determined using the NaOH melting-molybdenum antimony colorimetric method. For Olsen P, it was assessed using an extraction method involving 0.05 mol/L HCl combined with 0.025 mol/L (1/2 H_2_SO_4_). The evaluation of available K was carried out through the CH_3_COONH_4_ extraction method, followed by flame photometry. Acquisition of available B used the method of hot water reflux extraction. In this study, available Zn was determined through the DTPA (diethylenetriaminepentaacetic acid; C_14_H_23_N_3_O_10_) extraction method, coupled with atomic absorption spectroscopy (AAS).

### 2.4 Meteorological and topographic data collection

The sampling points of the fruit samples coincided with those of the soil samples. The latitude and longitude information were localized and recorded by GPS at the time of sampling so that each sampling point had location information. Data on the temperature and precipitation for 2022 were sourced from the National Tibetan Plateau Science Data Center (Peng, 2024a; Peng, 2024b). Annual average temperature and annual average precipitation were obtained by overlay analysis and the values were extracted to individual sampling points. This made each sampling point meteorologically informative. The 250-meter resolution DEM was obtained from the Geospatial Data Cloud. The DEM of the study area was obtained by mask extraction and thus calculating the slope, making each sampling point with topographic information.

### 2.5 Data processing and statistical analysis

The annual average temperature, annual average precipitation, and DEM were extracted to sampling points using ArcGIS 10.2. Soil, meteorological, and topographic factors along with fruit quality traits were used as independent and target variables, respectively. The descriptive analysis of fruit qualities in relation to meteorological and topographic conditions was conducted using Origin 2022 v.9.9.0.225 SR1 (Learning Edition). The correlations between fruit qualities and environmental variables were calculated and plotted using R 4.3.1. The number of principal components representing the dependent variable and their percentage contributions were calculated using R 4.3.1, followed by VIP calculations performed with SPSS 22. Multiple linear regression models were constructed using SPSS 22 to analyze the relationships between environmental factors and fruit qualities. The optimized linear programming method was implemented using LINGO 15.0, providing a theoretical basis for navel orange development. All tabular data were organized and classified using Excel 2016.

## 3 Results

### 3.1 Basic information of fruit qualities and environmental factors

#### 3.1.1 Basic information of fruit qualities

Within the core production area of the Gannan navel orange, the quality of navel oranges varied significantly among different orchards. In various orchards, the range of navel orange single fruit weight varied from 214.39 g to 518.19 g, with the maximum weight being 2.42 times the minimum. The coefficient of variation (CV) of single fruit weight was 16%, which is a moderate variation. The CV for transverse and vertical diameters were 5% and 6%, respectively. The minimum values of both diameters were approximately 74 mm, and the maximum values differed by no more than 3 mm. However, the mean value of vertical diameters was significantly larger than that of horizontal diameters. The fruit shape index of navel oranges ranged from 0.86 to 1.04, indicating the presence of both flat oval and long oval fruits among the sampled fruits (**Table 1**).

**Table 1:** Survey data of fruit qualities.

Fruit quality	Measuring item	Max	Min	Mean	STD	CV
fruit shape	fruit weight (g)	518.19	214.39	308.95	48.20	0.16
	horizontal diameter (mm)	100.05	74.30	83.56	4.05	0.05
	vertical diameter (mm)	102.74	74.78	88.51	5.69	0.06
	shape index	1.04	0.86	0.95	0.03	0.04
fruit peel	L*	73.37	60.20	68.91	2.33	0.03
	a*	23.04	-6.19	12.91	6.97	0.54
	b*	54.62	38.49	47.45	3.40	0.07
	H	1.72	1.11	1.31	0.14	0.10
fruit flavor	TSS (%)	13.17	8.73	10.66	0.93	0.09
	TA (%)	2.14	0.41	1.23	0.42	0.34
	TSS/TA	27.65	5.04	10.02	4.53	0.45
	Vitamin C (%)	61.29	25.51	40.54	7.92	0.20

L* represents luminance. a* represents green-red difference. b* represents yellow-blue difference.

The H value represents the hue angle of the pericarp’s color tone. The CV for L* is 3%, indicating minimal variability in peel brightness among navel oranges. Similarly, the b* and H values of navel oranges exhibited little difference, both indicating a low degree of variability. In contrast, the CV for a* reached 54%, signifying a medium level of variation and a high degree of variability in the red-green color difference among the collected navel oranges (**Table 1**).

Similarly, the TSS content ranged from 8.73% to 13.17%, with the maximum being 1.51 times the minimum. The TA content ranged from 0.41% to 2.14%, with the maximum value being 5.22 times the minimum. The TSS/TA, derived from dividing TSS by TA, varied with the maximum ratio being 5.49 times the minimum among different orchards. The CV indicates the relative dispersion of the data (Jalilibal et al., 2021). The CV for TSS content was 9%, indicating a low degree of variability and a more concentrated data distribution. Conversely, the CV for TA content and the TSS/TA content were 34% and 45%, respectively, suggesting high variability and more discrete data distribution. The range of Vitamin C content varied from 25.51% to 61.29%, indicating significant variation between the maximum and minimum values. Additionally, the CV for Vitamin C content was 20%, indicating a moderate level of variability (**Table 1**).

#### 3.1.2 Basic information of environmental factors

Environmental factors influence the growth and development of navel oranges, encompassing topographic, meteorological, and soil factors. Annual average precipitation ranged from 1634.80 mm to 1855.20 mm across different orchards, with a CV of 3%. Similarly, the annual average temperature ranged from 18.93°C to 20.93°C, with a CV of 2%, indicating concentrated data distribution. Among the 99 sampled orchards, elevations ranged from 161 m to 524 m, slopes from 0.34° to 29.11°. **Table 2** illustrates that elevation and slope exhibit high variability, reflecting significant data dispersion.

**Table 2:** Survey data of meteorological factors and topographic factors.

Measuring item	Max	Min	Mean	STD	CV
annual average precipitation (mm)	1855.20	1634.80	1752.61	56.63	0.03
annual average temperature (°C)	20.93	18.93	20.23	0.38	0.02
elevation (m)	524.00	161.00	275.61	85.97	0.31
slope (°)	29.11	0.34	9.64	5.95	0.62

The CV of the soil factors were all greater than 10% in the navel orange orchards in the core production area of the Gannan navel orange, which implies that these data have different degrees of dispersion. The result, as indicated in **Table 3**, showed that pH varies from 3.70 to 7.50, clay (1.96-11.64%), SOM (6.88-44.64 g/kg), Hydrolyzed N (28.60-155.00 mg/kg), total P (0.14-3.49 g/kg), Olsen P (0.37-231.25 mg/kg), CEC (3.11-14.12 cmol/kg), available K (59.00-497.00 mg/kg), available B (0.08-0.88 mg/kg), available Zn (0.36-41.30 mg/kg), exchangeable Ca (0.01-16.30 cmol/kg), exchangeable Mg (0.10-4.00 cmol/kg) with a CV of 15%, 31%, 37%, 70%, 82%, 32%, 40%, 52%, 146%, 37%, 111%, and 94%, respectively. They all have medium or high levels of dispersion.

**Table 3:** Survey data of soil factors.

Measuring item	Max	Min	Mean	STD	CV
pH	7.50	3.70	4.84	0.75	0.15
clay (%)	11.64	1.96	4.72	1.46	0.31
SOM (g·kg^-1^)	44.64	6.88	21.33	8.05	0.37
hydrolyzed N (mg·kg^-1^)	155.00	28.60	78.60	29.25	0.37
total P (g·kg^-1^)	3.49	0.14	0.72	0.51	0.70
Olsen P (mg·kg^-1^)	231.25	0.37	65.72	54.44	0.82
CEC (cmol·kg^-1^)	14.12	3.11	6.98	2.26	0.32
available K (mg·kg^-1^)	497.00	59.00	245.41	98.78	0.40
available B (mg·kg^-1^)	0.88	0.08	0.46	0.21	0.52
available Zn (mg·kg^-1^)	41.30	0.36	3.39	4.98	1.46
exchangeable Ca (cmol·kg^-1^)	16.30	0.01	2.66	2.94	1.11
exchangeable Mg (cmol·kg^-1^)	4.00	0.10	0.65	0.61	0.94

### 3.2 Determining the environmental factors effect on fruit qualities

#### 3.2.1 Determining the meteorological factors affecting fruit qualities

The annual average precipitation and annual average temperature were used as boundaries to assess the differences in fruit qualities under the influence of individual meteorological variables.

**Figure 2:**
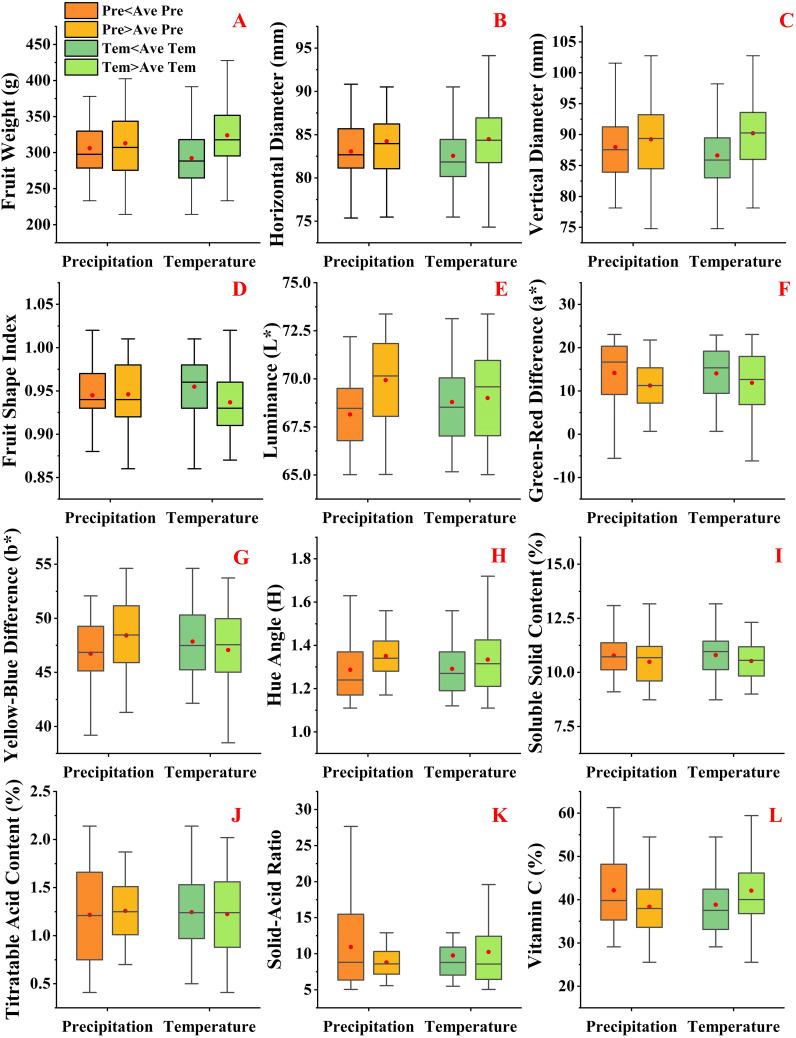
Changes in fruit qualities under meteorological condition **(A-D)** represent changes in fruit shape. **(E-H)** represent changes in fruit peel. **(I-L)** represent changes in fruit flavor.

Increasing annual precipitation led to greater diversity in fruit shape (**Figures 2A–D**). When precipitation exceeded the average, the upper limit of single fruit weight and vertical diameter increased, while the lower limit decreased. The upper and lower limits of the fruit shape index both decreased. This indicates that precipitation significantly impacted single fruit weight, vertical diameter, and fruit shape index. However, precipitation had no significant effect on horizontal diameter. Temperature significantly affected fruit shape (**Figures 2A–D**). Higher temperatures significantly increased the upper limits of single fruit weight, horizontal and vertical diameters, and expanded the range of horizontal diameters. Overall, the values of single fruit weight, horizontal diameter, and vertical diameter were greater when temperatures exceeded the average.

Both annual average precipitation and annual average temperature significantly affected changes in the fruit peels (**Figures 2E–H**). When precipitation increased, the upper limits of L* and b* rose, and the range of L* values increased. However, increased precipitation significantly raised the lower limit of a* and decreased the upper limit of H. This indicates that changes in average annual precipitation alter the color of the fruit peel. When temperature increased, the upper limits of L*, a*, and b* did not change significantly, nor did the lower limit of H. Meanwhile, the ranges of L* and b* remained large. Considering only annual average precipitation and annual average temperature, the change in TSS content was not significant, but the change in the TSS/TA was significant (**Figures 2I–L**). The upper limit of the TSS/TA decreased significantly with increased precipitation. Conversely, with increased temperature, the upper limit of the TSS/TA increased significantly.

#### 3.2.2 Determining the topographic factors affecting fruit qualities

**Figure 3:**
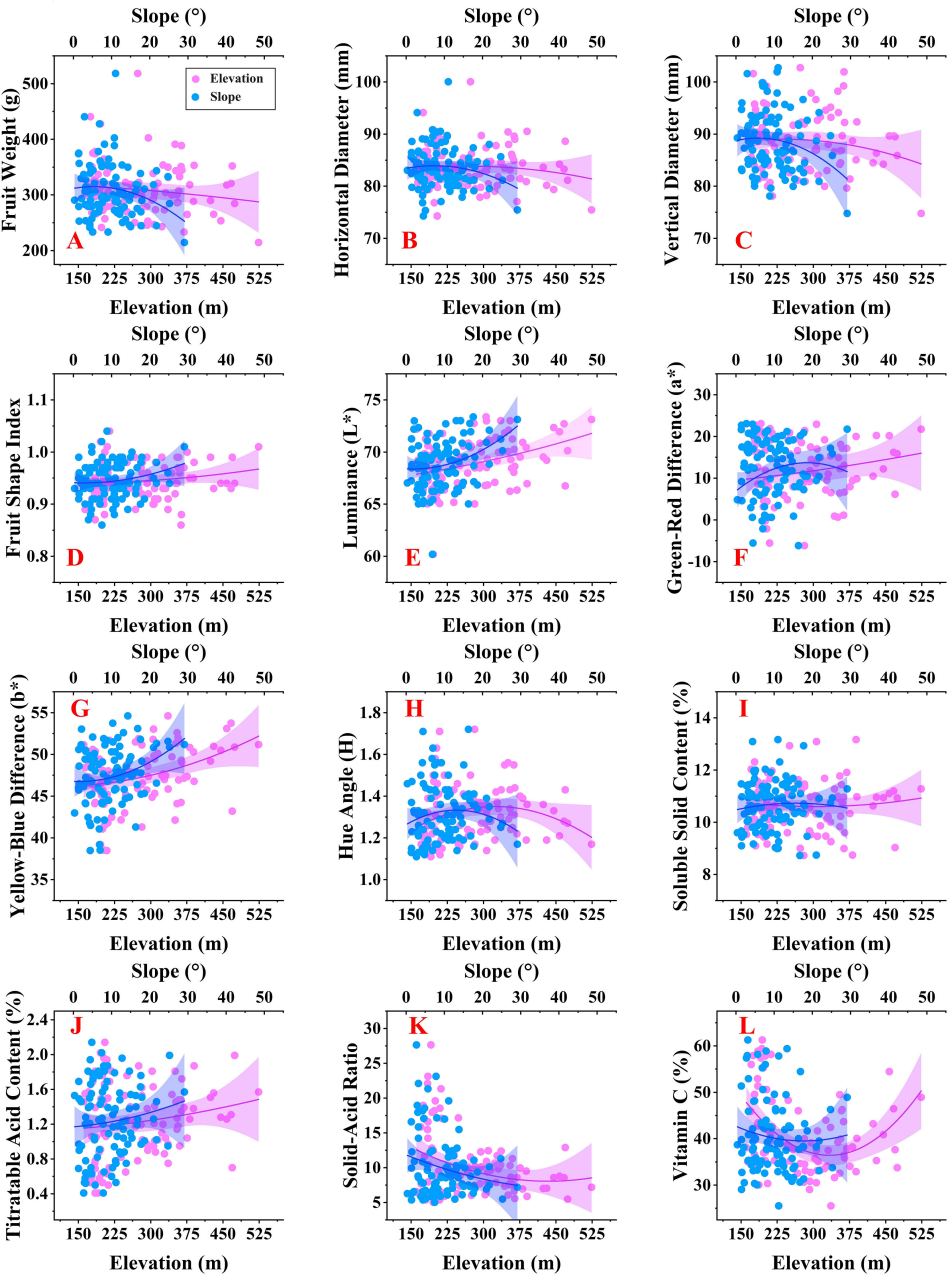
Changes in fruit qualities under topographic condition **(A-D)** represent changes in fruit shape. **(E-H)** represent changes in fruit peel. **(I-L)** represent changes in fruit flavor.

A second-order polynomial was utilized to model the relationship between fruit qualities and topographic factors. Most fruit quality traits were observed at elevations between 175m and 375m and slopes ranging from 0° to 20°. (**Figure 3**). Slope had a greater effect on fruit shape than elevation (**Figures 3A–D**). Single fruit weight, horizontal diameter, and vertical diameter decreased with increasing slope, indicating that steeper slopes were unfavorable for the accumulation of single fruit weight and fruit growth. Topographic conditions of low altitude and low slope were more conducive to producing navel oranges with standardized shapes.

Topographic conditions with high elevation and steep slopes were more likely to produce navel oranges with brighter coloration (**Figures 3E–H**). Increasing elevation promoted increases in L*, a*, and b* while increasing slope enhanced fruit brightness and the yellow-blue color difference. This demonstrates that both higher elevations and steeper slopes are beneficial in promoting a brighter, more yellow coloration of navel oranges. However, as elevation and slope increase, the hue angle (H) of the fruit skin decreases, which may prevent navel oranges from exhibiting a uniform yellow coloration.

Slope had little effect on fruit flavor, but elevation significantly influenced fruit flavor (**Figures 3I–L**). The TSS content remained constant across different slopes and elevations, indicating that these topographic factors did not significantly affect soluble solids. The TA content rose with higher slope and elevation, leading to a decrease in the TSS/TA. This suggests that high elevations and steep slopes are not suitable for producing navel oranges with a sweeter taste. However, high elevation and steep topographic conditions may be more suitable for Vitamin C accumulation (**Figure 3, L**).

#### 3.2.3 Determining the main soil factors affecting fruit qualities

**Figure 4:**
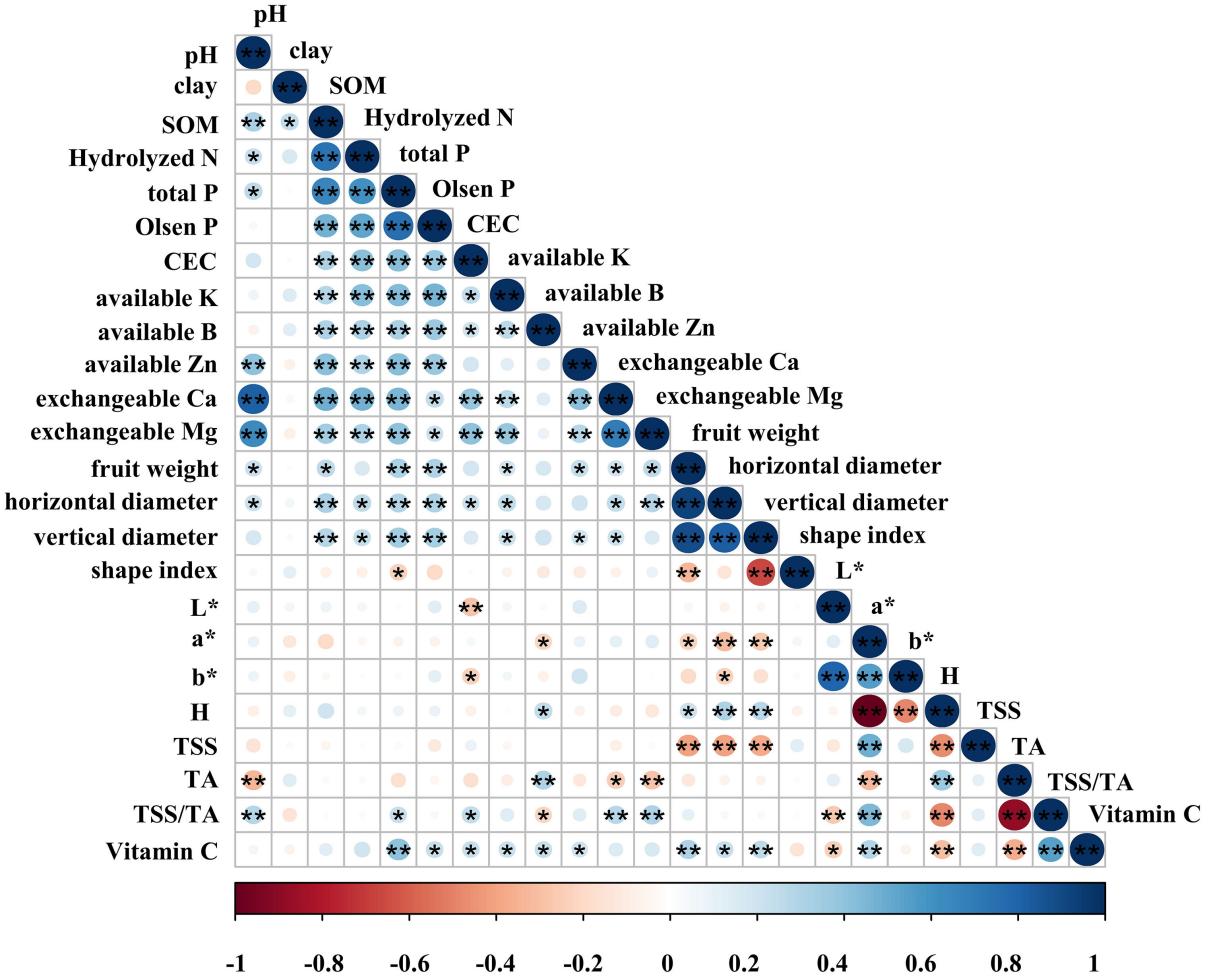
Correlation between environmental factors and fruit qualities “**” indicates that the correlation is significant at the 0.01 level, and “*” indicates that the correlation is significant at the 0.05 level.

The correlation coefficient was utilized to ascertain the direction and magnitude of the influence of soil factors on fruit qualities. Soil factors have a mutual influence on each other (**Figure 4**). The pH exhibited a highly significant positive correlation with SOM, available Zn, exchangeable Ca, and exchangeable Mg. Additionally, it showed a significant positive correlation with total P and Hydrolyzed N. Clay was significantly and positively correlated with SOM. SOM has a significant positive correlation with clay and highly significant positive correlations with other soil factors. There is no statistically significant correlation between Hydrolyzed N and clay, but Hydrolyzed N shows a significant positive correlation with pH. Additionally, Hydrolyzed N has a highly significant positive correlation with other soil factors. The situation of Total P is similar to that of Hydrolyzed N; it is not significantly correlated with clay, but Total P has a highly significant positive correlation with other soil factors. Olsen P has no significant correlation with pH and clay, but it has a significant correlation with exchangeable Ca and exchangeable Mg, and it has a highly significant positive correlation with CEC, available K, available B, available Zn, and Hydrolyzed N. CEC showed a significant positive correlation with available K and available B. Additionally, it showed a highly significant positive correlation with Hydrolyzed N, exchangeable Ca, and exchangeable Mg. Available K exhibited a highly significant positive correlation with available B, Hydrolyzed N, exchangeable Ca, and exchangeable Mg. Available B exhibited a highly significant positive correlation with Hydrolyzed N. Available Zn exhibited a highly significant positive correlation with Hydrolyzed N, exchangeable Ca, and exchangeable Mg. Hydrolyzed N exhibited a highly significant positive correlation with exchangeable Ca and exchangeable Mg. Additionally, it showed a highly significant positive correlation with exchangeable Ca and exchangeable Mg. Correlations were observed among other environmental elements; however, these correlations were not statistically significant.

Correlation coefficients allow for determining the direction and magnitude of soil factors’ influence on fruit qualities (**Figure 4**). Fruit shapes were primarily influenced by meteorological and soil factors. Topographic factors had minimal influence on fruit shapes. Fruit weight exhibited a highly significant positive correlation with total P and Olsen P. Additionally, it showed a significant positive correlation with pH, SOM, available K, available Zn, exchangeable Ca, and exchangeable Mg. Horizontal diameter exhibited a highly significant positive correlation with SOM, total P, Olsen P, and exchangeable Mg. It showed a significant positive correlation with pH, Hydrolyzed N, CEC, available K, and exchangeable Ca. Vertical diameter exhibited a highly significant positive correlation with SOM, total P, and Olsen P. Additionally, it showed a significant positive correlation with Hydrolyzed N, available K, available Zn, and exchangeable Ca. The shape index exhibited a significant negative correlation with total P.

Soil factors have a minimal impact on fruit peels. L* exhibited a highly significant negative correlation with CEC. The a* showed a significant negative correlation with available B. The b* showed a significant negative correlation with CEC. The value of H exhibited a significant positive correlation with available B. Soil factors had a minor influence on TSS content, but their correlation was not statistically significant. The correlation between some soil factors and TA content was robust. The TA content exhibited a highly significant negative correlation with pH and exchangeable Mg. Besides, it showed a significant negative correlation with exchangeable Ca. Additionally, it showed a highly significant positive correlation with available B. Soil factors primarily have a positive effect on TSS/TA. The TSS/TA exhibited a significant positive correlation with total P and CEC. Besides, it showed a highly significant positive correlation with pH, exchangeable Ca, and exchangeable Mg. Additionally, it showed a significant negative correlation with available B. Soil factors have a positive effect on Vitamin C. Vitamin C exhibited a highly significant positive correlation with total P and had a significant positive correlation with Olsen P, CEC, available K, available B, and available Zn.

### 3.3 Multivariate analysis of the relationship between fruit qualities and environmental factors

#### 3.3.1 Establishing multiple regression equations

PLSR (Partial Least Squares Regression) is the product of a combination of PCA, CCA, and multiple linear regression (Höskuldsson, 1988). With PLSR, the VIP (Variable Importance for the Projection) can be calculated. Variables with a VIP score over 1.0 are generally considered to be particularly important to the model (Abdi, 2010). In this study, PLSR was used to determine the significance of each variable and the number of principal components, and VIP values were used to screen out significant variables. Also, this study used variables with VIP>1.0 to construct multiple regression equations (**Table 4**).

**Table 4:** Regression equation of environmental factors affecting fruit qualities.

Objective functions ^a^	Affecting factors ^b^	Regression equation	*F*	*p*
Y_1_	C_2_, S_3_, S_5_, S_6_, S_10_, S_12_	Y_1_=-471.633 + 36.999C_2_+0.637S_3_-3.499S_5_+0.184S_6_+0.93S_10_+9.681S_12_	4.35	0.001
Y_2_	S_3_, S_4_, S_5_, S_6_, S_8_, S_11_, S_12_	Y_2_ = 79.618-0.136S_3_-0.014S_4_-0.84S_5_+0.018S_6_+0.003S_8_+0.008S_11_+1.222S_12_	2.78	0.012
Y_3_	C_2_, S_3_, S_4_, S_5_, S_6_	Y_3_ = 0.126 + 4.154C_2_+0.115S_3_+0.002S_4_+0.341S_5_+0.023S_6_	5.11	<0.001
Y_4_	C_2_, T_1_, T_2_, S_5_, S_6_	Y_4_ = 1.278-0.016C_2_-0.00002T_1_+0.001T_2_-0.011S_5_-0.0001S_6_	2.34	0.047
Y_5_	C_1_, C_2_, T_1_, T_2_, S_7_	Y_5_ = 7.421 + 0.004C_1_+2.596C_2_+0.013T_1_+0.058T_2_-0.205S_7_	8.09	<0.001
Y_6_	C_1_, S_3_, S_9_, S_12_	Y_6_ = 69.033-0.03C_1_-0.104S_3_-5.659S_9_+1.777S_12_	4.34	0.003
Y_7_	C_1_, T_1_, T_2_, S_7_, S_10_	Y_7_ = 51.316-0.003C_1_+0.01T_1_+0.061T_2_-0.283S_7_+0.129S_10_	4.06	0.002
Y_8_	C_1_, C_2_, T_1_, S_3_, S_9_	Y_8_= -0.901 + 0.001C_1_+0.01C_2_+0.0004T_1_+0.001S_3_+0.098S_9_	4.49	0.001
Y_9_	S_1_, S_9_, S_11_, S_12_	Y_9_ = 1.577-0.112S_1_+0.612S_9_+0.001S_11_-0.133S_12_	6.29	<0.001
Y_10_	C_1_, T_1_, S_1_, S_5_, S_7_, S_11_, S_12_	Y_10_ = 53.934-0.032C_1_+0.003T_1_+2.009S_1_+2.123S_5_+0.234S_7_-0.418S_11_+0.725S_12_	4.45	<0.001
Y_11_	T_1_, S_5_, S_6_, S_7_, S_8_, S_9_	Y_11_ = 38.014-0.016T_1_+6.711S_5_-0.02S_6_+0.071S_7_+0.004S_8_+3.977S_9_	4.39	<0.001

^a^Y_1_~Y_11_ denote fruit weight, horizontal diameter, vertical diameter, shape index, Luminance (L*), Green-Red Difference (a*), Yellow-Blue Difference (b*), Hue Angle (H), titratable acid (TA) content, solid-acid ratio (TSS/TA), Vitamin C, respectively. ^b^C_1_~C_2_ denote annual average precipitation, and annual average temperature, respectively; T_1_~T_2_ denote elevation, and slope, respectively; S_1_ ~ S_12_ denote pH, clay, SOM, Hydrolyzed N, total P, Olsen P, cation exchange capacity (CEC), available K, available B, available Zn, exchangeable Ca, exchangeable Mg, respectively.

**Figure 5:**
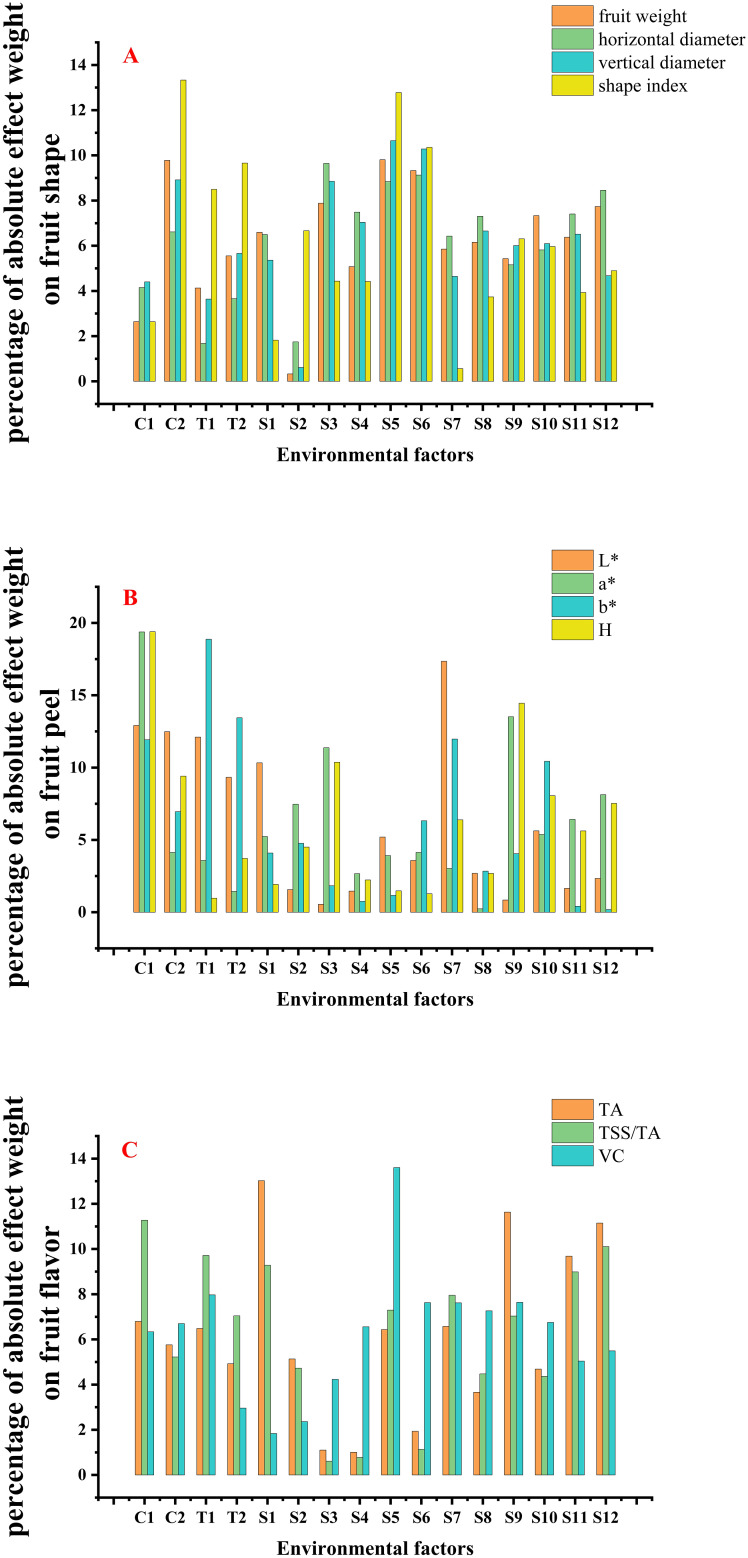
Percentage of the importance of factors on fruit qualities **(A–C)** represent the percentage of importance of different factors on fruit shape, fruit peel, and fruit flavor, respectively. C_1_~C_2_ denote annual average precipitation, and annual average temperature, respectively; T_1_~T_2_ denote elevation, and slope, respectively; S_1_~S_12_ denote pH, clay, SOM, Hydrolyzed N, total P, Olsen P, cation exchange capacity (CEC), available K, available B, available Zn, exchangeable Ca, exchangeable Mg, respectively.

In this study, the model’s contribution percentage highlighted the significance of meteorological, topographic, and soil factors on fruit qualities in the core production area of Gannan navel oranges (**Figure 5**). Among these factors, soil had the greatest impact on fruit shape, surpassing the influence of both topographic and meteorological variables (**Figure 5A**). C_2_, S_3_, S_5_, S_6_, S_10_, and S_12_ exhibited higher influence on fruit weight, with their absolute weight values contributing 9.78%, 7.88%, 9.81%, 9.32%, 7.33%, and 7.74%, respectively. The cumulative contribution of these soil factors reached 42.08%. The fruit’s horizontal diameter was primarily influenced by soil factors, with S_3_, S_4_, S_5_, S_6_, S_8_, S_11_, and S_12_ contributing 9.64%, 7.49%, 8.84%, 9.13%, 7.40%, and 8.46%, respectively. The cumulative contribution of these factors amounted to 50.96%. C_2_, S_3_, S_4_, S_5_, and S_6_ contributed 8.91%, 8.85%, 7.04%, 10.65%, and 10.28% to the vertical diameter of the fruits, respectively. The cumulative contribution of these soil factors amounted to 36.82%, whereas the meteorological factors contributed only 8.91%. C_2_, T_1_, T_2_, S_5_, and S_6_ contributed 13.33%, 8.50%, 9.66%, 12.78%, and 10.36% to the fruit shape index, respectively. The cumulative contribution of topographic factors was 18.16%, while soil factors contributed 23.14%. The cumulative contribution of soil factors to fruit weight, horizontal diameter, and vertical diameter was significantly greater than that of meteorological and topographic factors. Although the cumulative contribution of soil factors to the fruit shape index was higher, the difference was not significant. This indicates that the fruit shape index is similarly influenced by topographic, meteorological, and soil factors.

Topographic, meteorological, and soil factors vary in their impact on fruit peel (**Figure 5B**). C_1_, C_2_, T_1_, T_2_, and S_7_ contributed 12.91%, 12.48%, 12.11%, 9.33%, and 17.35% to the value of L*, respectively. The cumulative contributions of meteorological and topographic factors were 25.39% and 21.44%, respectively. C_1_, S_3_, S_9_, and S_12_ contributed 19.38%, 11.37%, 13.52%, and 8.12% to the value of a*, respectively. The cumulative contribution of soil factors was 33.01%. C_1_, T_1_, T_2_, S_7_, and S_10_ contributed 11.92%, 18.87%, 13.44%, 11.98%, and 10.44% to the value of b*, respectively. The cumulative contributions of topographic and soil factors were 32.31% and 22.42%, respectively. C_1_, C_2_, T_1_, S_3_, and S_9_ contributed 19.40%, 9.41%, 0.97%, 10.37%, and 14.45% to the value of H, respectively. The cumulative contributions of meteorological and soil factors were 28.81% and 24.82%, respectively. The L* and b* are influenced by various factors, including topography, meteorology, and soil. Specifically, L* is primarily affected by meteorological factors, while b* is predominantly influenced by topographic factors. In contrast, a* and H are mainly affected by soil and meteorological factors, with minimal impact from topographic factors.

Fruit flavor was significantly influenced by soil factors (**Figure 5C**). The contributions of S_1_, S_9_, S_11_, and S_12_ to TA were 13.02%, 11.63%, 9.68%, and 11.14%, respectively. The cumulative contribution of soil factors was 45.47%. C_1_, T_1_, S_1_, S_5_, S_7_, S_11_, and S_12_ contributed 11.27%, 9.72%, 9.28%, 7.30%, 7.95%, 8.99%, and 10.11% to TSS/TA, respectively. The cumulative contribution of soil factors was 43.63%. T_1_, S_5_, S_6_, S_7_, S_8_, and S_9_ contributed 7.97%, 13.60%, 7.63%, 7.62%, 7.27%, and 7.64% to vitamin C, respectively. The cumulative contribution of soil factors was 43.76%. The TA is predominantly influenced by soil factors, which have the most significant impact. Besides, the TSS/TA is significantly affected by meteorological, topographic, and some soil factors. For vitamin C, soil factors also exert a greater influence than topographic factors.

#### 3.3.2 Optimizing environmental factors for high-quality Gannan navel

In this study, utilizing VIP, the factors significantly affecting fruit qualities were identified, and a multiple regression model based on fruit quality indices was developed (**Table 4**). This model also passed significance testing, indicating the model’s stability and reliability.

To further investigate the range of environmental factors that optimize the fruit quality of Gannan navel oranges, the linear regression equation (**Table 4**) is employed. It set the maximum value of a specific fruit qualities index of navel oranges as the objective function, with other fruit qualities indices and environmental factors as constraints. Thus, a linear programming equation was established to determine the optimal fruit quality index. When calculating the maximum value of a specific fruit quality factor, it is essential to ensure that the other fruit quality factors also achieve high standards. A specific range of limits was imposed on the environmental factors in the calculation process. In this study, the lower constraint limit of the index of fruit qualities was the average value of the sampled fruits, and the upper constraint limit of the environmental factor was the maximum value of the sampled orchards.

Taking the calculation of the maximum weight of a single fruit as an example, this study constructed a system of linear programming equations for Gannan navel orange fruit quality and environmental factors:


$$\begin{array}{l} \text{MAX }\begin{array}{l} Y_{1}=-471.633+36.999C_{2}+0.637S_{3}-3.499S_{5}+0.184S_{6}+0.93S_{10}+9.681S_{12} \end{array};\\ \begin{array}{l} Y_{2}=79.618-0.136S_{3}-0.014S_{4}-0.84S_{5}+0.018S_{6}+0.003S_{8}+0.008S_{11}+1.222S_{12} \end{array}\geq 83.56;\\ \begin{array}{l} Y_{3}=0.126+4.154C_{2}+0.115S_{3}+0.002S_{4}+0.341S_{5}+0.023S_{6} \end{array}\geq 88.51;\\ \begin{array}{l} Y_{4}=1.278-0.016C_{2}-0.00002T_{1}+0.001T_{2}-0.011S_{5}-0.0001S_{6} \end{array}\leq 0.95;\\ \begin{array}{l} Y_{5}=7.421+0.004C_{1}+2.596C_{2}+0.013T_{1}+0.058T_{2}-0.205S_{7} \end{array}\geq 68.91;\\ \begin{array}{l} Y_{6}=69.033-0.03C_{1}-0.104S_{3}-5.659S_{9}+1.777S_{12} \end{array}\geq 12.91;\\ \begin{array}{l} Y_{7}=51.316-0.003C_{1}+0.01T_{1}+0.061T_{2}-0.283S_{7}+0.129S_{10} \end{array}\geq 47.45;\\ Y_{8}=-0.901+0.001C_{1}+0.01C_{2}+0.0004T_{1}+0.001S_{3}+0.098S_{9}\geq 1.31;\\ \begin{array}{l} Y_{9}=1.577-0.112S_{1}+0.612S_{9}+0.001S_{11}-0.133S_{12} \end{array}\leq 1.23;\\ Y_{10}=53.934-0.032C_{1}+0.003T_{1}+2.009S_{1}+2.123S_{5}+0.234S_{7}-0.418S_{11}+0.725S_{12}\geq 10.02;\\ \begin{array}{l} Y_{11}=38.014-0.016T_{1}+6.711S_{5}-0.02S_{6}+0.071S_{7}+0.004S_{8}+3.977S_{9} \end{array}\geq 40.54; \end{array}$$

where, 1752.61≤C_1_ ≤ 1855.20, 20.23 ≤ C_2_ ≤ 20.93, 275.61 ≤ T_1_ ≤ 524.00, 9.64 ≤ T_2_ ≤ 29.11, 4.84 ≤ S_1_ ≤ 7.50, 4.72 ≤ S_2_ ≤ 11.64, 21.33 ≤ S_3_ ≤ 44.64, 78.60 ≤ S_4_ ≤ 155.00, 0.72 ≤ S_5_ ≤ 3.49, 65.72 ≤ S_6_ ≤ 231.25, 6.98 ≤ S_7_ ≤14.12, 245.41 ≤ S_8_ ≤ 497.00, 0.46 ≤ S_9_ ≤ 0.88, 3.39 ≤ S_10_ ≤ 41.30, 2.66 ≤ S_11_ ≤ 16.30, 0.65≤ S_12_ ≤ 4.00.

Applying the same method, linear programming equations can be established, and they can be used to calculate the maximum horizontal diameter, maximum vertical diameter, minimum shape index, maximum L*, maximum a*, maximum b*, maximum H, minimum TA, maximum TSS/TA, and maximum Vitamin C. When each quality index of navel orange was optimized (Y_1_ = 441.79 g, Y_2_ = 85.65 mm, Y_3_ = 96.77 mm, Y_4_ = 0.89, Y_5_ = 76.25, Y_6_ = 18.74, Y_7_ = 56.43, Y_8_ = 1.48, Y_9_ = 0.49%, Y_10_ = 25.44, Y_11_ = 54.87%), the optimum environmental factors in the core production area of the Gannan navel orange were: annual average precipitation 1752.61-1855.20 mm, annual average temperature 20.93 °C, elevation 275.61-524.00 m, slope 9.64-29.11°, pH 7.50, clay 11.64%, SOM 21.33-36.73 g/kg, Hydrolyzed N 78.60 mg/kg, total P 0.72-2.89 g/kg, Olsen P 136.02-231.25 mg/kg, CEC 6.9 -14.12 cmol/kg, available K 497.00mg/kg, available B 0.46-0.88 mg/kg, available Zn 41.30 mg/kg, exchangeable Ca 2.66-16.30 cmol/kg, exchange Mg 4.00 cmol/kg (**Table 5**).

**Table 5:** Optimum schemes of environmental factors for fruit qualities^a^.

Affecting factors ^b^	Y_1_	Y_2_	Y_3_	Y_4_	Y_5_	Y_6_	Y_7_	Y_8_	Y_9_	Y_10_	Y_11_	Range of optimum affecting factors
C_1_ (mm)	1768.49	1783.89	1770.70	1752.61	1855.20	1752.61	1752.61	1855.20	1825.05	1752.61	1783.89	1752.61-1855.20
C_2_ (°C)	20.93	20.93	20.93	20.93	20.93	20.93	20.93	20.93	20.93	20.93	20.93	20.93
T_1_ (m)	275.61	275.61	275.61	524.00	524.00	456.70	524.00	524.00	275.61	524.00	275.61	275.61-524.00
T_2_ (°)	9.64	9.64	9.64	9.64	29.11	9.64	29.11	9.64	9.64	9.64	9.64	9.64-29.11
S_1_	7.50	7.50	7.50	7.50	7.50	7.50	7.50	7.50	7.50	7.50	7.50	7.50
S_2_ (%)	11.64	11.64	11.64	11.64	11.64	11.64	11.64	11.64	11.64	11.64	11.64	11.64
S_3_ (g·kg^-1^)	36.73	21.33	34.52	21.33	21.33	21.33	21.33	24.95	21.33	21.33	21.33	21.33-36.73
S_4_ (mg·kg^-1^)	78.60	78.60	78.60	78.60	78.60	78.60	78.60	78.60	78.60	78.60	78.60	78.60
S_5_ (g·kg^-1^)	0.76	0.76	0.76	2.89	1.50	1.20	1.50	1.19	0.72	2.76	2.89	0.72-2.89
S_6_ (mg·kg^-1^)	231.25	231.25	231.25	231.25	172.21	152.38	172.21	179.38	136.02	231.25	231.25	136.02-231.25
S_7_ (cmol·kg^-1^)	14.12	14.12	14.12	14.12	6.98	14.12	6.98	14.12	14.12	14.12	14.12	6.98-14.12
S_8_ (mg·kg^-1^)	497.00	497.00	497.00	497.00	497.00	497.00	497.00	497.00	497.00	497.00	497.00	497.00
S_9_ (mg·kg^-1^)	0.88	0.88	0.88	0.46	0.46	0.46	0.46	0.88	0.46	0.46	0.88	0.46-0.88
S_10_ (mg·kg^-1^)	41.30	41.30	41.30	41.30	41.30	41.30	41.30	41.30	41.30	41.30	41.30	41.30
S_11_ (cmol·kg^-1^)	16.30	16.30	16.30	16.30	2.66	16.30	2.66	16.30	2.66	2.66	16.30	2.66-16.30
S_12_ (cmol·kg^-1^)	4.00	4.00	4.00	4.00	4.00	4.00	4.00	4.00	4.00	4.00	4.00	4.00

^a^Y_1_~Y_11_ denote fruit weight, horizontal diameter, vertical diameter, shape index, Luminance (L*), Green-Red Difference (a*), Yellow-Blue Difference (b*), Hue Angle (H), titratable acid (TA) content, solid-acid ratio (TSS/TA), Vitamin C, respectively.

^b^C_1_ ~ C_2_ denote annual average precipitation, and annual average temperature, respectively; T_1_ ~ T_2_ denote elevation, and slope, respectively; S_1_ ~ S_12_ denote pH, clay, SOM, Hydrolyzed N, total P, Olsen P, cation exchange capacity (CEC), available K, available B, available Zn, exchangeable Ca, exchangeable Mg, respectively.

## 4 Discussion

### 4.1 Relationship between fruit qualities of Gannan navel orange and environmental factors

Each environmental factor influenced different fruit qualities. In this study, we analyzed the relationship between topographic factors (T_1_-T_2_), meteorological factors (C_1_-C_2_), soil factors (S_1_-S_12_), and fruit quality factors to obtain the weights of environmental factors on fruit quality (**Table 4**).

Fruit shapes were significantly influenced by meteorological conditions and soil properties (Aular et al., 2017). The annual average temperature (C_2_) had varying effects on fruit shape. As C_2_ increased, both fruit weight and vertical diameter grew. This is probably because the annual average temperature in the core production area of Gannan navel oranges ranges from 18.93°C to 20.93°C. When the temperature is higher, it approaches the ideal range for navel orange growth and development (Fernández-Echeverría et al., 2024). Therefore, a higher annual average temperature is more suitable for fruit growth within the appropriate temperature range (Nawaz et al., 2020). However, excessively high temperatures caused an imbalance between the horizontal and vertical diameters, leading to fruit cracking (Kanayama and Kochetov, 2015). Additionally, the exchangeable Mg (S_12_) had a significant positive effect on fruit weight and horizontal diameter. This finding is consistent with Andrews and Brathwaite (2006) research on the relationship between navel orange yield and soil nutrient concentration.

The color of the fruit peels was significantly influenced by various environmental factors such as meteorological, topographic, and soil conditions (Rodrigo et al., 2013). C_1_ simultaneously affected L*, a*, b*, and H, which indicated that precipitation impacts the fruit epidermis (Nawaz et al., 2021). Elevation (T_1_) affected both L* and b*. As T_1_ increased, temperature decreased, affecting L* and b*, which demonstrated that temperature influences color (Rodrigo et al., 2013).

Environmental factors had a minor but insignificant effect on TSS content (Gupta et al., 2022). The result as indicated in **Figure 2** showed that the average TSS content of navel orange pulp slightly decreased with increasing temperature, which is consistent with the findings of Mesejo et al. (2024), but the specific effect of temperature on TSS remains unclear (Onwude et al., 2024). 60% of the variation in TSS content could be attributed to the fruit weight (Castle, 1995). From **Figure 4**, TSS content was significantly negatively correlated with fruit weight, horizontal diameter, and vertical diameter. While it is not possible to identify a statistically significant environmental factor affecting TSS content, it is evident that the relationship between TSS content and fruit shape is closely linked (Xin et al., 2022). The pH (S_1_) and exchangeable Mg (S_12_) had a negative effect on TA content. This is consistent with the results of Wu et al (2020), who studied the effect of Biochar on fruit qualities of mandarin. In contrast, the most significant positive effect on TA content was observed with available B. The TA content increased with an increase in available B (Walli et al., 2022). Additionally, the TA content is significantly higher when Zn + B is applied Zhang et al. (2015). Moreover, B also promotes the accumulation of fruit weight (Boaretto et al., 2011).

From **Figure 4**, available B exhibited a significant negative effect on TSS/TA. Increasing the soil content of available B promoted the accumulation of TSS in fruits (Turhan, 2021). Sajid et al. (2024) demonstrated that Boron enhances the TSS/TA of fruit pulps, leading to improved yield and quality. Additionally, annual average precipitation (C_1_), slope (T_1_), pH (S_1_), total P (S_5_), CEC (S_7_), exchangeable Ca (S_11_), and exchangeable Mg (S_12_) influenced the TSS/TA in fruits. Among them, total P (S_5_) had the most significant positive effect on the value of TSS/TA. An increase in soil total P content corresponded to an increase in the TSS/TA. This is in agreement with the results of Su et al. (2024).

Topographic, meteorological, and soil factors each exert varying degrees of influence on vitamin C content (Comunian et al., 2022). When the annual average temperature exceeds 20.23°C, the conditions become favorable for vitamin C synthesis in navel oranges (Lee and Kader, 2000). Additionally, navel oranges accumulate more vitamin C when the annual average precipitation is below 1,752.61 mm, compared to environments with higher precipitation (Hussain et al., 2017). From **Table 4**, total P (S_5_) and available B (S_9_) had significant positive effects on vitamin C, consistent with findings from previous studies (Luo et al., 2023).

### 4.2 Optimum environmental factors for high-quality Gannan navel orange

Navel orange cultivation considers factors such as topography, climate, and soil, all of which influence fruit qualities. Additionally, orchard management practices play a crucial role (Bakhshandeh et al., 2022; Guo et al., 2023).

The growth of navel orange requires an annual average precipitation of more than 1000 mm and the suitable annual average precipitation is 1000-1500 mm (Mesejo et al., 2024). In the core production area of the Gannan navel orange, the annual average precipitation ranged from 1752.61 to 1855.20 mm, which approached the upper limit of suitable levels. The average temperature required to induce flowering in navel oranges is below 24°C, with a minimum growth temperature of 13°C (Zabihi et al., 2015). Aloisi et al. (2020) and Agustí et al. (2022) mentioned that the suitable temperature for citrus plants ranges from 15 to 23°C, and the optimal annual average temperature in the present study area is 20.93°C.

Additionally, optimal topographic factors included an elevation of 275.61-524.00 m and a slope of 9.64-29.11°. Citrus planting should ideally occur at elevations below 500 m, as lower elevation areas are more conducive to citrus growth (Wu et al., 2021). Some studies found a preference for navel orange orchards in lower elevation areas (Tercan and Dereli, 2020; Mokarram and Mirsoleimani, 2022). In this study, the upper limit of the optimal elevation exceeded 500 meters, likely because higher elevations enhance the brightness of fruit color (Gómez-Devia and Nevo, 2024). As a result, the optimal elevation determined through linear programming surpassed 500 meters. Zabihi et al. (2019) identified that areas with a slope of 15° are suitable for citrus planting.

Oranges thrive in soils with a pH ranging from 4 to 9, however, pH levels exceeding 8 are generally considered unsuitable for cultivating navel oranges (Li et al., 2020). Citrus orchards with high yields have SOM greater than 15 g/kg, and in some orchards, it ranges from 20 to 60 g/kg (Ahmad et al., 2022). The study by Barlas and Kadyampakeni (2022) found that high yields of navel oranges occurred when the total P content in the soil exceeded 0.4 g/kg. Cheng et al. (2015) examined the soil characteristics of high-yield and high-quality navel orange orchards, indicating that orchards with a CEC of 5.08 to 35.83 cmol/kg and clay content of 6.76% to 48.40% are suitable for navel orange cultivation. An Olsen P range of 100 to 200 mg/kg indicates moderate soil nutrient content, while available K > 300 mg/kg and available Zn > 10 mg/kg signify soil richness in nutrients (Hippler et al., 2015; Yan et al., 2022; Wu et al., 2021). Wen et al. (2022) identified that optimal soil nutrient ranges for citrus plantings include exchangeable Ca ranging from 1.94 to 12.47 cmol/kg, exchangeable Mg from 0.58 to 1.8 cmol/kg, effective B between 0.74 and 1.10 mg/kg, and Hydrolyzed N from 144.3 to 271.7 mg/kg. These findings underscore the importance of maintaining specific soil nutrient levels to ensure the healthy growth and development of citrus plants.

## 5 Conclusion

This study focuses on the core production area of Gannan navel oranges, investigating the relationship between fruit quality and various environmental factors. The results indicated that soil factors exert a more substantial impact on fruit quality. Climate factors also significantly influence both the yield and quality of navel oranges, while topographic factors primarily affect the fruit peel. Different attributes of fruit quality exhibit varying responses to these environmental factors. Through the use of multiple regression models and linear programming equations, the optimal environmental conditions for the growth of Gannan navel oranges have been identified.

## Data Availability

The original contributions presented in the study are included in the article/supplementary material. Further inquiries can be directed to the corresponding authors.
